# Thoracic and Umbilical Endometriosis Concurrently Presenting During Secondary Subfertility Evaluation

**DOI:** 10.1002/rcr2.70760

**Published:** 2026-09-22

**Authors:** Nabeela Khan, Saadia Ashraf, Hamza Khan Khattak, Afra Khan, Kamil Ahmad Kamil

**Affiliations:** ^1^ Department of Pulmonology Khyber Teaching Hospital Peshawar Pakistan; ^2^ Khyber Medical College Peshawar Pakistan; ^3^ Internal Medicine Department Nasrat Bashir Curative Hospital Kandahar Kandahar Province Afghanistan

**Keywords:** pleural effusion, pleural endometriosis, subfertility, thoracic endometriosis syndrome, umbilical endometriosis

## Abstract

Thoracic endometriosis is a rare manifestation of extra pelvic endometriosis and it is often missed because of its variable clinical presentation. A 33‐year‐old woman undergoing evaluation for secondary subfertility presented with a three‐year history of cyclical periumbilical pain, swelling and occasional catamenial bleeding. Contrast‐enhanced CT (CECT) abdomen and pelvis demonstrated an umbilical soft tissue nodule consistent with umbilical endometriosis which was histologically confirmed. Incidentally this CECT also revealed a right‐sided pleural effusion with a pleural‐based nodule despite absence of respiratory symptoms. Thoracentesis yielded haemorrhagic exudative fluid, while thoracoscopy showed multiple nodules and haemorrhagic cysts on parietal and visceral pleura. Histopathology and immunohistochemistry confirmed pleural endometriosis. The patient subsequently developed a pneumothorax requiring chest tube repositioning and was treated with gonadotropin‐releasing hormone (GnRH) analogue therapy, resulting in complete symptomatic and radiological resolution at follow‐up. Hormonal therapy was later discontinued to pursue conception; however, recurrence of pleural effusion occurred, necessitating continuation of the medication. This case highlights the silent and multifocal nature of extra‐pelvic endometriosis.

## Graphical abstract

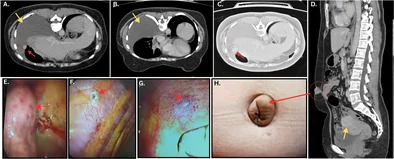

## 1 Introduction

Endometriosis is defined by the presence of endometrial‐like glands and stroma outside the uterine cavity. Patients commonly present with chronic pelvic pain, infertility and painful or heavy menstrual periods [1]. The condition affects approximately 5%–15% of women of reproductive age and is found in up to 70% of women with chronic pelvic pain. While the ovaries are the sites most frequently involved, about 12% of patients have extra‐genital endometriosis, most commonly located within the abdominopelvic region. Rarely, endometriosis can extend beyond the abdomen to involve the pleura, pericardium and central nervous system [2]. Ectopic endometrial tissue within the lung parenchyma or pleura often remains under‐recognized and is diagnosed late due to its asymptomatic course [3].

We report a case of a 33‐year‐old woman who presented for evaluation of secondary subfertility and pelvic pain and was incidentally found to have concurrent umbilical and pleural endometriosis discovered incidentally on CECT chest and the patient had no respiratory symptoms. Previous reports have described concurrent umbilical and thoracic endometriosis, although the clinical presentations have varied. Elsayed et al. reported a patient with an umbilical endometriotic lesion and recurrent, asymptomatic right‐sided pleural effusion, with thoracoscopic biopsy confirming pleural endometriosis [3]. Other reports have described thoracic endometriosis but without abdominal wall involvement presenting as catamenial haemorrhagic pleural effusion [4, 5].

## 2 Case Report

**FIGURE 1:**
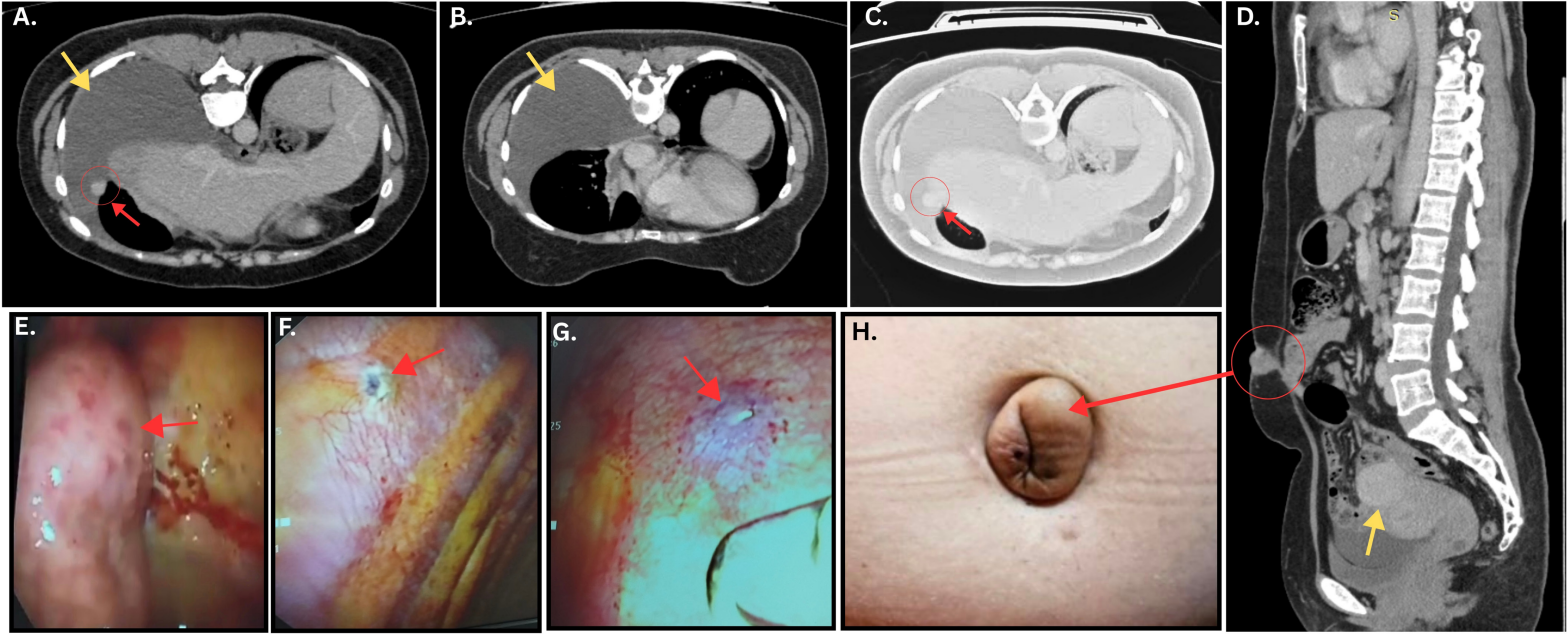
Radiologic, thoracoscopic and clinical findings of concurrent thoracic and umbilical endometriosis. (A, B) Contrast‐enhanced CT images of the lower thorax demonstrating a 1.2 × 1.1 cm pleural‐based nodule along the diaphragmatic pleural surface (red arrows) with associated moderate right‐sided pleural effusion (yellow arrows). (C) HRCT lung window. (D) CT scan of the abdomen and pelvis revealing an umbilical soft‐tissue nodule measuring 2.1 × 2.0 × 2.3 cm (red arrows) and a 5.6 × 4.8 cm subserosal fundal fibroid (yellow arrow). (E–G) Thoracoscopic images demonstrating multiple haemorrhagic cystic lesions scattered over the hyperemic parietal pleura (red arrows). (H) Photograph showing an enlarged umbilical swelling on physical examination.

A 33‐year‐old multiparous woman with a BMI of 22, married for 9 years and mother to an alive 8‐year‐old child, presented for evaluation of secondary subfertility. She reported a 3‐year history of cyclical periumbilical pain associated with a progressively enlarging umbilical swelling (shown in Figure 1H) that became more prominent and tender during menstruation, with occasional catamenial bleeding. She had previously undergone an exploratory laparotomy for these symptoms; however, no definitive diagnosis had been established at that time. However, the patient denied respiratory symptoms such as cough, pleuritic chest pain or dyspnoea.

She had no known history of chronic respiratory disease, pulmonary tuberculosis, diabetes mellitus, hypertension, connective tissue disease or previous malignancy. She is a non‐smoker with no significant occupational or environmental exposures. There was no family history of endometriosis, infertility, gynaecological malignancy or chronic pulmonary disease. Her menstrual history included a regular menstrual cycle. Her age at menarche was 12 years, cycle length was 30 days, duration of menstrual flow was 7 days with severe dysmenorrhea with history of increase in size of umbilical swelling with pain during each cycle since last 3 years. Her obstetric history was gravida 1, para 1 (G1P1). Her gynecologic history was further significant for a failed in vitro fertilization (IVF) attempt 1 year prior to presentation.

On physical examination, the patient appeared comfortable and was not in respiratory distress. Vitally, she was stable with a respiratory rate of 16/min and oxygen saturation of 97% on room air. On examination of the chest, expansion was reduced on the right side. Percussion revealed dullness over the right lower hemithorax, while auscultation demonstrated reduced breath sounds over the right lower lung hemithorax without added sounds. Cardiovascular and abdominal examinations were otherwise unremarkable except for the tender umbilical swelling.

On initial evaluation, ultrasound of the umbilical region demonstrated features suggestive of endometriosis. A contrast‐enhanced CT scan of the chest, abdomen and pelvis (Figure 1A–D) was performed, which revealed an umbilical soft tissue nodule. Mild ascites and a lobulated uterus with multiple fibroids, including a 5.6 × 4.8 cm subserosal fundal fibroid, were also noted. Unexpectedly, sections of the lower thorax demonstrated a small pleural‐based nodule along the diaphragmatic pleural surface with moderate right‐sided pleural effusion. The rest of the viscera was normal. She was referred to the pulmonology ward for further evaluation.

Chest ultrasound estimated the pleural fluid volume at approximately 1200 mL. Diagnostic thoracentesis yielded grossly haemorrhagic fluid. Laboratory analysis revealed an exudative lymphocyte‐predominant profile (75% lymphocytes) with an albumin level of 5 g/dL. Pleural fluid adenosine deaminase (ADA) was 42.1 IU/L. Cytology was negative for malignant cells and culture showed no bacterial growth.

**FIGURE 2:**
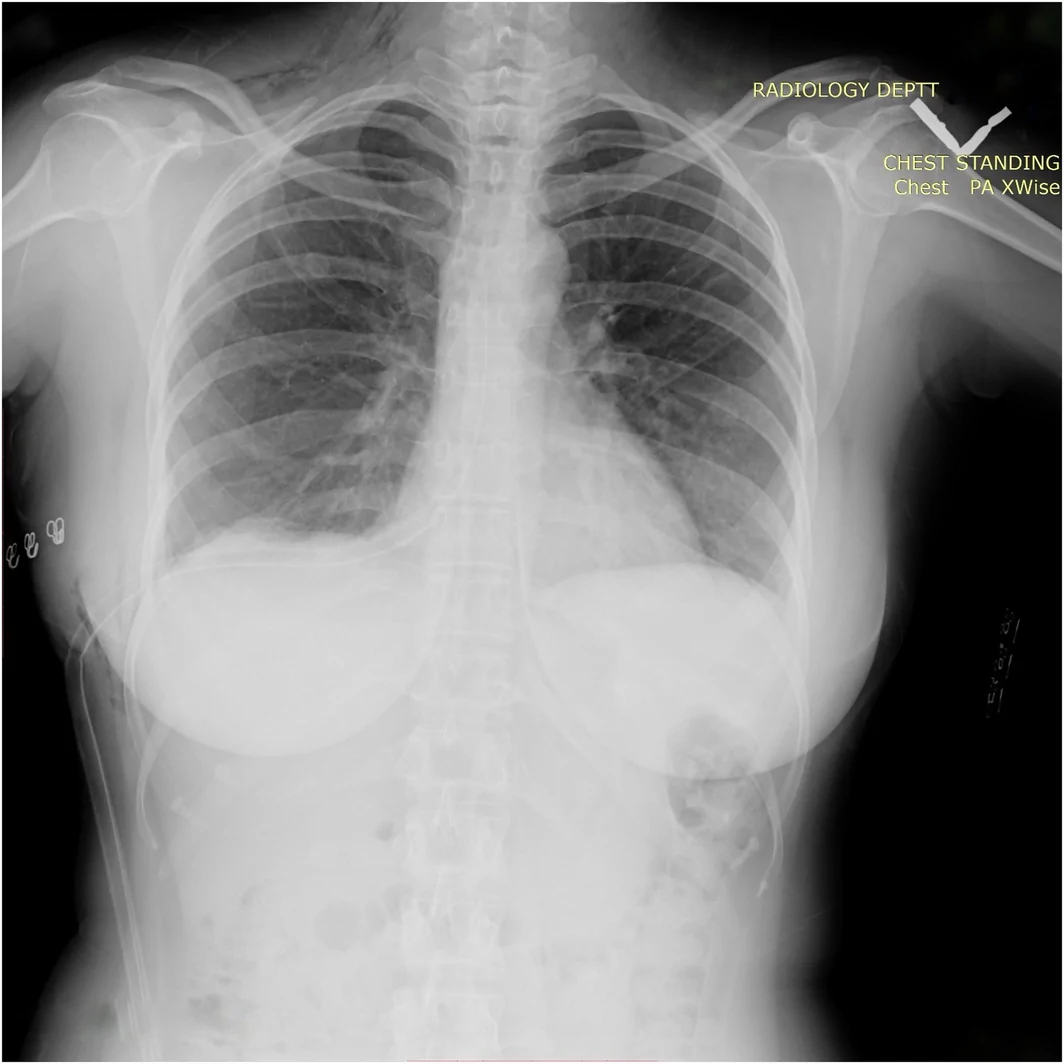
Chest radiograph after right‐sided chest tube insertion.

Given the radiologic and pleural fluid findings, diagnostic thoracoscopy was performed. Thoracoscopic evaluation showed multiple yellow, firm nodules and haemorrhagic cysts scattered across a hyperemic parietal pleura (Figure 1E–G). The visceral pleura was also abnormal, and the right lung was partially collapsed. A chest tube was inserted, and subsequent Chest X‐ray imaging (shown in Figure 2) confirmed re‐expansion of the lung, although the tube swing remained persistently positive.

Histopathological examination of the pleural biopsy specimen revealed fibroconnective tissue containing endometrial‐type glands with surrounding pseudodecidualized stroma and abundant hemosiderin‐laden macrophages, findings consistent with ectopic endometrial tissue involvement. Immunohistochemistry supported the diagnosis, with PAX8 positivity in glandular epithelium indicating Müllerian origin and CD10 highlighting stromal cells. Iron stain confirmed hemosiderin deposition, while p40 was negative, excluding squamous or mesothelial malignancy. Overall, findings were consistent with pleural endometriosis when correlated with clinical and radiological features. With the availability of the histopathology report, the differentials of tuberculous pleural effusion, malignant pleural effusion and pleural metastasis were excluded.

One week later, a repeat high‐resolution CT scan showed a pneumothorax, requiring repositioning of the chest tube. The patient was discharged with the chest tube in situ and remained asymptomatic from any respiratory complaints. She was referred to a gynaecologist who started her on hormonal suppression therapy with a gonadotropin‐releasing hormone (GnRH) analogue after discussing with her all the treatment options available. At the three‐month follow‐up, the umbilical swelling and associated para‐umbilical pain had completely resolved, with no evidence of pleural effusion on repeat imaging.

Hormonal therapy was later temporarily discontinued as the patient wished to pursue conception; however, recurrence of the pleural effusion was noted during follow‐up, prompting re‐initiation of GnRH analogue therapy, to which she again responded favourably with sustained symptomatic improvement. Overall, the patient responded to treatment with marked symptomatic and radiological resolution.

## 3 Discussion

Thoracic endometriosis is a rare form of extragenital endometriosis in which endometrial tissue implants on the pleura, lung parenchyma or diaphragm. Several theories have attempted to explain its pathogenesis, including retrograde menstruation with transdiaphragmatic passage, haematogenous or lymphatic microembolization, coelomic metaplasia and stem cell differentiation. However, no single mechanism fully accounts for all cases [6].

Thoracic endometriosis may present clinically as thoracic endometriosis syndrome (TES), which includes catamenial pneumothorax, catamenial hemothorax, catamenial hemoptysis and pulmonary nodules. Reported frequencies vary, with catamenial pneumothorax being the most common (73%), followed by catamenial hemothorax (14%), catamenial haemoptysis (7%) and pulmonary nodules (6%) [7]. As many as 70% of cases may be asymptomatic. When symptoms occur, they typically coincide with menses—starting approximately 1 day before onset of flow and lasting up to 3 days after. Symptoms commonly include chest or scapular pain, dyspnoea, haemoptysis and occasionally cough.

Recognized predictors of TES include age greater than 31 years, coexisting pelvic endometriosis, right‐sided thoracic involvement and non‐smoking status [7]. This preferential right‐sided involvement is linked to the favourable flow of peritoneal fluid from the right paracolic gutter to the right sub‐phrenic space combined with congenital or acquired fenestrations in the right hemidiaphragm that facilitate trans‐diaphragmatic migration. The protective effect of the heart and pericardium on the left side may further contribute to this asymmetry [6]. Different theories such as retrograde menstruation, haematogenous or lymphatic dissemination, coelomic metaplasia and stem cell differentiation support the extra‐pelvic spread of endometrial cells [8, 9]. The coexistence of umbilical and pleural disease in our patient may reflect multifocal dissemination through lymphatic or haematogenous pathways in addition to transdiaphragmatic spread, although no single mechanism adequately explains all cases.

Diagnostic evaluation of TES relies on a combination of imaging and invasive assessment. Chest radiography can detect pneumothorax, pleural effusions, consolidation and nodules. CT scans provide better lesion localization, identifying ground‐glass opacities, consolidation, nodules, cystic lesions or pneumothorax. MRI helps characterize haemorrhagic implants and soft tissue lesions. Bronchoscopy may reveal endobronchial bleeding but often fails to detect peripheral or pleural lesions. Video‐assisted thoracoscopic surgery (VATS) is considered the gold standard for diagnosing and treating TES, especially in cases of catamenial pneumothorax or pleural disease [6, 10].

Histological confirmation requires identifying endometrial glands and stroma on haematoxylin and eosin staining. Hemosiderin‐laden macrophages are frequently present due to recurrent bleeding. Immunohistochemistry with markers such as oestrogen receptor (ER), progesterone receptor (PR) and CD10 helps confirm the diagnosis, especially when glandular structures are sparse [8, 9].

Management of thoracic endometriosis is individualized according to the patient's clinical presentation, severity of disease and reproductive goals. Acute stabilization for pneumothorax or pleural effusion follows standard thoracic management with needle decompression or chest tube placement [11]. Long‐term management comprises medical therapy to suppress ectopic endometrial activity and includes GnRH analogues, combined oral contraceptives, progestins and aromatase inhibitors. However, as such treatment only suppresses ectopic implants, recurrence following treatment discontinuation is common. VATS remains the gold standard surgical management of TES, allowing excision of endometriotic lesions. It is recommended for intolerance, contraindications or failure of medical therapy, recurrent symptoms, diaphragmatic repair and pleurodesis or when definitive diagnosis and treatment are necessary [12, 13]. Literature review showed that the recurrence rates were 50% and 60% after medical management as compared to the 5% and 25% after surgical management at 6 and 12 months respectively. Combined surgical and postoperative hormonal suppression has also been used to reduce the risk of recurrence [12].

In our patient, pleural endometriosis was confirmed histologically and she was clinically stable, with no persistent pneumothorax requiring immediate surgical intervention. All the treatment options available including VATS, pleural excision and pleurodesis, were discussed with the patient. No diaphragmatic abnormality was identified on CECT or during thoracoscopy; therefore, diaphragmatic repair was not considered necessary. Patient opted for medical therapy and was therefore referred to gynaecologist who started her on GnRH analogue therapy as the initial treatment. As she was also being evaluated for secondary subfertility and wished to conceive, the implications of hormonal suppression for fertility were also discussed. GnRH therapy was subsequently discontinued when she wished to pursue pregnancy. However, the recurrence of pleural effusion after stopping treatment, followed by improvement after restarting GnRH therapy, suggested that medical treatment was suppressing rather than eradicating the disease.

Given her underlying subfertility and desire for future pregnancy, continued follow‐up with gynaecology and reproductive medicine is important. IVF may be considered once thoracic endometriosis resolves and hormonal therapy is stopped; however, it is associated with chances of recurrence of the disease.

Fertility outcomes in thoracic endometriosis are dependent on the coexistence of pelvic endometriosis, which is reported in 50%–84% of cases and is a well‐recognized cause of sub‐fertility. Medical therapy helps with controlling disease but does not fully eradicate it and results in difficulty in conception. VATS with laparoscopy is the preferred therapeutic management in patients who require future pregnancy [14]. Therefore, a multidisciplinary team involving gynaecology, thoracic surgery, pulmonology and radiology is essential for effective management of the disease and future fertility outcomes.

Concurrent thoracic and umbilical endometriosis should be considered as a manifestation of multifocal endometriosis, particularly during subfertility evaluation. Early diagnosis, multidisciplinary coordination and timely hormonal or surgical management can help prevent complications and optimize outcomes, particularly in women undergoing fertility evaluation.

In conclusion, thoracic endometriosis may be completely asymptomatic. Histopathology remains the diagnostic gold standard. Recurrence is common after discontinuation of hormonal therapy. Early multidisciplinary management is essential to effectively treat the disease, minimize recurrence and address future fertility goals.

VATS remains the gold standard for diagnosis and surgical management when tissue confirmation or definitive treatment is required.

## Author Contributions

Nabeela Khan contributed to data collection, patient history documentation and drafting of the initial manuscript. Saadia Ashraf assisted in the clinical management of the patient and contributed to the interpretation of diagnostic findings. Hamza Khan Khattak and Afra Khan participated in the literature review, manuscript editing and formatting of the report. Kamil Ahmad Kamil supervised the study, critically revised the manuscript for important intellectual content and provided final approval for submission. All authors reviewed and approved the final version of the manuscript and agree to be accountable for its content.

## Ethics Statement

This case report was conducted in accordance with the principles of the Declaration of Helsinki. Ethical approval for the study was reviewed and granted by the Institutional Review Ethics Board (IREB) of Khyber Medical College, chaired by Dr. Farooq Ahmed (Approval No. 1117/DME/KMC).

## Consent

The authors declare that written informed consent was obtained for the publication of this manuscript and accompanying images and attest that the form used to obtain consent from the patient complies with the Journal requirements as outlined in the author guidelines.

## Conflicts of Interest

The authors declare no conflicts of interest.

## Data Availability

The data that support the findings of this study are available on request from the corresponding author. The data are not publicly available due to privacy or ethical restrictions.
